# Evaluation of the revised Nipissing District Developmental Screening (NDDS) tool for use in general population samples of infants and children

**DOI:** 10.1186/s12887-016-0577-y

**Published:** 2016-03-16

**Authors:** John Cairney, Jean Clinton, Scott Veldhuizen, Christine Rodriguez, Cheryl Missiuna, Terrance Wade, Peter Szatmari, Marilyn Kertoy

**Affiliations:** Department of Family Medicine, McMaster University, 175 Longwood Road South, Suite 109A, Hamilton, ON L8P 0A1 Canada; Offord Centre for Child Studies, McMaster University, Hamilton, ON Canada; CanChild Centre for Childhood Disability Research, McMaster University, Hamilton, ON Canada; Department of Psychiatry and Behavioral Neurosciences, McMaster University, Hamilton, ON Canada; Centre for Addiction and Mental Health, Health Systems Research and Consulting Unit, Toronto, ON Canada; School of Rehabilitation Sciences, McMaster University, Hamilton, ON Canada; Department of Community Health Sciences, Brock University, St Catharines, ON Canada; Department of Psychiatry, University of Toronto, Toronto, ON Canada; Child, Youth and Family Program, Centre for Addiction and Mental Health, Toronto, ON Canada; School of Communication Sciences and Disorders, Western University, London, ON Canada

## Abstract

**Background:**

There is widespread interest in identification of developmental delay in the first six years of life. This requires, however, a reliable and valid measure for screening. In Ontario, the 18-month enhanced well-baby visit includes province-wide administration of a parent-reported survey, the Nipissing District Developmental Screening (NDDS) tool, to facilitate early identification of delay. Yet, at present the psychometric properties of the NDDS are largely unknown.

**Method:**

812 children and their families were recruited from the community. Parents (most often mothers) completed the NDDS. A sub-sample (*n* = 111) of parents completed the NDDS again within a two-week period to assess test-retest reliability. For children 3 or younger, the criterion measure was the Bayley Scales of Infant Development, 3rd edition; for older children, a battery of other measures was used. All criterion measures were administered by trained assessors. Mild and severe delays were identified based on both published cut-points and on the distribution of raw scores. Sensitivity, specificity, positive and negative predictive values were calculated to assess agreement between tests.

**Results:**

Test-retest reliability was modest (Spearman’s rho = .62, *p* < 001). Regardless of the age of the child, the definition of delay (mild versus severe), or the cut-point used on the NDDS, sensitivities (from 29 to 68 %) and specificities (from 58 to 88 %) were poor to moderate.

**Conclusion:**

The modest test-retest results, coupled with the generally poor observed agreement with criterion measures, suggests the NDDS should not be used on its own for identification of developmental delay in community or population-based settings.

## Background

The first six years of life are the crucial period of human development, and there is broad consensus that investment in optimizing health and development in this period will result in significant individual, social and economic benefits [1]. Results from developmental neuroscience suggest that both prevention and treatment efforts need to occur as early in this period as possible, as treatment later in life may be less effective in preventing poor outcomes [2, 3].

Developmental delay is one target for early identification and intervention. While the prevalence of global delay in children under 6 is between 1 and 3 % [4], 12 to 16 % of children show meaningful delay in one or more cognitive, motor, language, and socio-emotional areas [5–7]. Such delays are associated with increased risk of future physical and mental health problems and with poor functional and educational outcomes later in life [8, 9].

Early intervention requires early identification. The detection rate of developmental delay in clinical settings, however, is well below the estimated prevalence [10]. Systematic screening provides a possible solution, but requires measures that are cost-effective, easily administered, reliable, and valid. These requirements are exacting, given the complexities of measuring development in early childhood [11]. While early screening and surveillance is recommended by many professional organizations [5, 10], and has been implemented in many countries, there is no consensus on the instruments to be used.

The Nipissing District Developmental Screening tool (NDDS), is increasingly used for this purpose in Canada [12, 13] and the United States (e.g., Early Head Start Program: http://www.nemcsa.org/headstart/ECDHS_A.aspx). The NDDS was first developed in 1993, and its content and design were revised in 2011. It comprises 13 age group-specific parent-completed checklists of developmental milestones for children between 1 month and 6 years of age. In Ontario, the NDDS is one of the recommended measures to be used during the recently-implemented enhanced 18-month well-baby visit [14, 15], a population-wide, comprehensive developmental assessment and parenting education session connected to the 18-month immunization visit. In Ontario, the government has paid to provide free access to the NDDS to all parents.

Despite its increasing use, the psychometric properties of the NDDS are largely unknown; we could locate only three reports, two of them unpublished, and all limited by small samples [16–18]. Only Currie et al. [16] evaluated the current version of the NDDS, and this was a pilot study of 31 children, only 4 of whom met criteria for mild developmental delay. The psychometric properties of the NDDS have not therefore been assessed with an adequate sample.

## Methods

### Sample

We recruited a sample of participants from community organizations who provide services to families in Hamilton, Ontario and surrounding areas and which targeted sociodemographically diverse populations. Organizations included Ontario Early Years Centres and Parent and Family Literacy Centres. Staff of some organizations shared information about the study with their clients, and some referred families directly. We also used recruitment posters and notices on web sites, and operated a booth at the Hamilton Baby and Toddler Expo, which is well-attended by families from Hamilton and surrounding areas. Families were recruited between May 2010 and October 2011. Parents were eligible if they could speak and read English, and were the child’s primary caregiver and legal guardian. We aimed to recruit 50 children for each of the NDDS’s 10 age bands up to 36 months (group A; *n* = 500) and 100 in each of the remaining 3 age bands (4 to 6 years of age; group B; *n* = 300), for a total of 800 children across all 13 age bands. Child age was adjusted for prematurity if the child was under 2 years and born 4 weeks or more prematurely.

### Study design

We randomly selected 111 (14 %) participants to complete the NDDS a second time after an interval of 2 weeks, and 55 (7 %) to complete a qualitative interview. Criterion measures were administered by research assistants, all of whom had an undergraduate or Master’s degree (e.g., psychology, health sciences). RAs received a minimum of 8 h of pre-test administration training and at least 10 h of supervised test administration experience prior to being able to conduct independent assessments. Assessment reports were monitored continuously for quality assurance throughout the study. We received ethical approval from the McMaster University Research Ethics Board, and all parents provided informed, written consent.

### Parent-completed measures

#### Nipissing district developmental screen-2011

The NDDS-2011 asks parents to indicate whether they have observed their child performing various motor, cognitive or language tasks. There are separate checklists for each of 13 age groups. The checklist for infants under 1 month old includes 4 items, while others include between 12 and 22 items. Milestones not yet observed by the caregiver are counted to produce a score. Current recommendations are for a health professional to follow up with any scores of 1 or higher. Before the 2011 revision, a cut-point of 2 or higher was used [12, 17]. As the proportion of children identified at the 1+ threshold may be too large for some situations, we also explored the performance of the NDDS at the 2+ cut-point.

### Criterion measures

As there is no single gold standard for assessing development in children, we designed a protocol using widely-used instruments with demonstrated reliability and validity. Given the broad age range covered by the NDDS, it was not possible to use the same criterion measure for all children. For children 3 years and under (Group A), we used the Bayley Scales of Infant Development, 3rd Edition (BSID-III; 19). The BSID-III produces a set of raw and normal scores for each of five domains: Cognition, receptive communication, expressive communication, fine motor, and gross motor. We identified as “mildly delayed” those children who scored below the “borderline” cut-point in one or more domains, and as “severely delayed” those with at least one score below the “extremely low” cut-point according the manual [19].

For children aged 4 to 6 (Group B), we selected three separate measures assessing development in motor coordination, cognition, and language: the Movement Assessment Battery for Children, 2nd Edition (M-ABC; 20); the Kaufman Brief Intelligence Test, 2nd Edition (KBIT-2) [20]; and the Pre-school Language Scale, 4th edition (PLS-4) [21, 22], respectively. The M-ABC [20], PLS-4 [21], and KBIT-2 [23] have all shown good agreement with clinical evaluation and with other instruments. Children were identified as having “mild” or “severe” delay by using the 15th and 5th percentile cut-points on each instrument. The M-ABC does not provide a 15th percentile cut-point; instead, the 16th percentile is recommended [20]. The K-BIT produces a standard score with a mean of 100 and an SD of 15. We therefore used cut-points of 84.5 and 75, which correspond to the 15th and 5th percentiles.

On the BSID-III, the published “borderline” cut-points produced a prevalence of 27 % in children under 1 and of only 5 % in those aged 2 or 3. It is unlikely that this reflects genuine variation within our sample, as we drew on the same sources to recruit all participants. Concerns over published BSID-III norms have also been raised previously [24]. We therefore produced a second set of classifications (i.e., cut-points to classify mild and severe delay) based on the distributions of raw scores. We repeated this process for the PLS-4, as the norms for this instrument identified only a single “case”. The K-BIT and M-ABC produced plausible prevalence’s, based on the literature, that did not vary markedly with child age.

To produce distribution-based indicators of caseness, we used quantile regression, with the scale score as the outcome and fractional polynomial transformations of age as the independent variables. These models yield equations that can be solved at any child age to calculate a cut-point at the designated quantile. For the BSID-III, we fit two models for the raw score of each subscale: One corresponding to the “borderline” (−1.33 SDs; 9.2nd percentile) and one to the “extremely low” (−2 SD; 2.275th percentile) cut-point. For the PLS-4, to be consistent with other measures used for older children, we estimated cut-points at the 5th and 15th percentiles. To do this analysis, we used the xmfp Stata program by Royston [25].

### Statistical analysis

We measured test-retest reliability by calculating Spearman correlations for total scores and kappa statistics for agreement using scores of 1 and 2 as cut-points.

We compared the NDDS with the criterion measures by calculating sensitivity, specificity, positive predictive value (PPV) and negative predictive value (NPV), along with exact binomial 95 % confidence intervals. We used Stata 13 for all analyses [26].

## Results

**Fig. 1:**
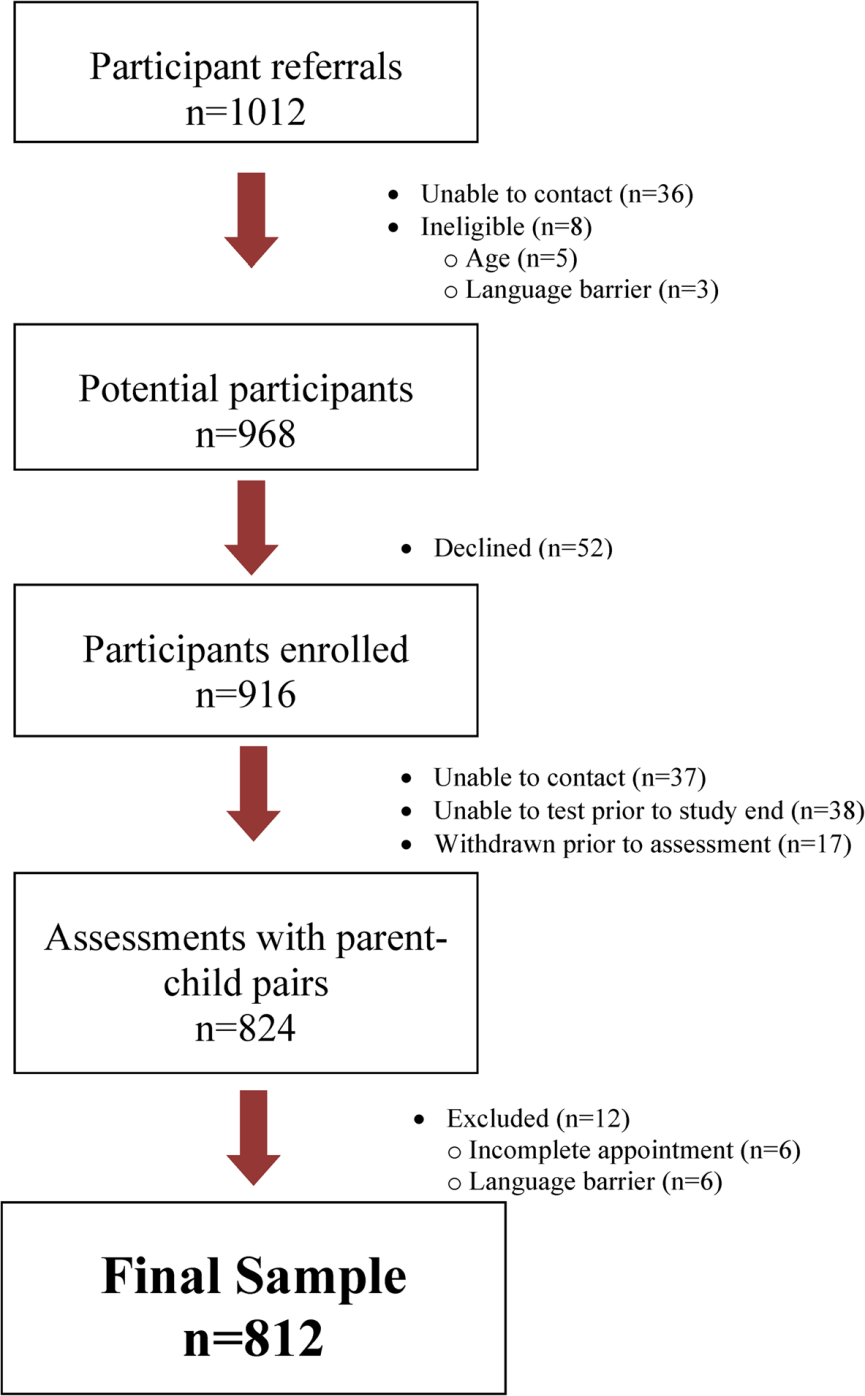
Participant flow diagram

We received initial referrals for 1012 parent–child pairs and have final data for 812: 594 children aged 1 month to 36 months (Group A) and 218 children aged 4 to 6 years (Group B). This represents an 80.2 % response rate from the total sample of referrals, and an 83.8 % response from eligible families. Figure 1 shows the stages of recruitment, participant exclusions, and consent rate. Parent demographics are shown in Table 1. In 98 % of cases, the NDDS was completed by the child’s biological mother, and the 812 child-parent pairings were drawn from 572 families. The number of children in each NDDS age band varied from 41 to 98.

**Table 1:** Sample Description

	Group A	Group B	Total
N	594	218	812
Sex of Person Most Knowledgeable			
Female	586 (99 %)	213 (98 %)	799 (99 %)
Male	8 (1 %)	4 (2 %)	12 (1 %)
Home ownership			
Rent	137 (23 %)	42 (20 %)	179 (22 %)
Own	449 (76 %)	171 (80 %)	620 (77 %)
Other (eg, lives with family)	3 (1 %)	1 (<1 %)	4 (<1 %)
Marital status			
Never married	32 (5 %)	6 (3 %)	38 (5 %)
Married, common-law, or living with a partner	545 (92 %)	202 (94 %)	747 (93 %)
Separated or divorced	14 (2 %)	8 (4 %)	22 (3 %)
Education			
Some secondary or less	25 (4 %)	5 (2 %)	30 (4 %)
Completed high school or GED	27 (5 %)	15 (7 %)	42 (5 %)
Some college or technical training	25 (4 %)	13 (6 %)	38 (5 %)
Completed college or technical training	130 (22 %)	45 (21 %)	175 (22 %)
Some university	40 (7 %)	16 (7 %)	56 (7 %)
Completed a bachelor’s degree (BA, BSc, etc.)	212 (36 %)	81 (37 %)	293 (36 %)
Completed a graduate or professional degree (MSc, MD, etc.)	135 (23 %)	42 (19 %)	177 (22 %)
Household income (2009)			
Under $35,000	75 (14 %)	28 (14 %)	103 (14 %)
$35,000 to $59,999	81 (15 %)	30 (15 %)	111 (15 %)
$60,000 to $89,999	115 (21 %)	41 (20 %)	156 (21 %)
$90,000 to $129,999	162 (29 %)	60 (30 %)	222 (29 %)
$130,000 or higher	118 (21 %)	43 (21 %)	161 (21 %)
Child’s sex			
Male	306 (52 %)	104 (48 %)	410 (51 %)
Female	288 (48 %)	113 (52 %)	401 (49 %)
Number of siblings (mean (SD))	0.9 (0.9)	1.3 (0.8)	1.0 (0.9)
Age of enrolled child in months (mean (SD))	31.2 (4.7)	30.2 (4.5)	30.9 (4.7)

### Test-retest reliability

Test-retest reliability after a two-week delay was moderate (Spearman’s rho = 0.61, *p* < 0.001), as was agreement at specific cut-points (at the 1+ cut-point, kappa = 0.59; 2+, kappa = 0.57). 86 of 111 (78 %) retests produced the same result as the initial screen; of the remainder, 15 (14 %) scores decreased and 10 (9 %) increased. The difference between the proportions increasing and decreasing was not significant (exact binomial *p* = 0.42).

### Criterion validity

**Fig. 2:**
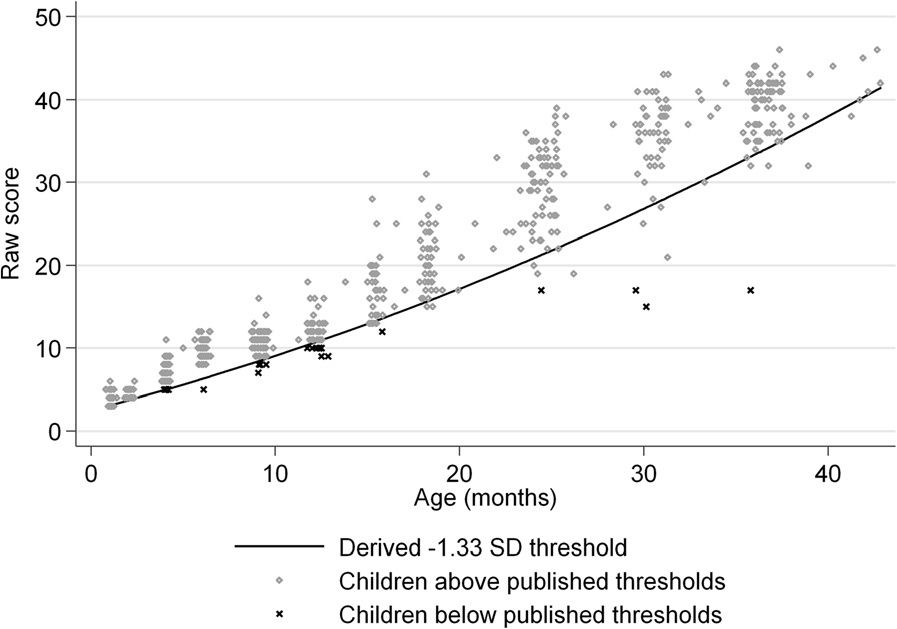
Cases and non-cases according to published norms for BSID-III expressive communication subscale, with distribution-based cut-point line derived from quantile regression

We fit models to identify distribution-based cut-points for the BSID-III and PLS-4. In both cases, these resulted in higher prevalence than those derived using the published norms, and in prevalences that did not vary substantially with child age. Results of this analysis are illustrated in Fig. 2, which shows ‘borderline’ cases on the expressive communication subscale of the BSID-III according to the published cut-points (crosses) and according to our distribution-based model (all those below the regression line). Similar results were obtained for the other BSID-III subscales and for the PLS-4.

#### Group A (children 1 month to 3 years of age)

103 of 594 children (17.3 %) scored in the “borderline” range in one or more BSID-III domains. At the recommended 1+ cut-point (i.e., one or more “no” responses on the NDDS), the sensitivity of the NDDS was 59 % and the specificity 67 %. 17 children (2.9 %) scored in the “extremely low” range in at least one domain, and the sensitivity and specificity in this case were 65 % and 63 %, respectively (see Table 2).

**Table 2:** Agreement between NDDS and BSID-III-based indicators of delay for children aged 3 and under (Group A; *n* = 594)

	Mild delay				Severe delay			
	Published norms		Distribution-based cut-points		Published norms		Distribution-based cut-points	
	1+	2+	1+	2+	1+	2+	1+	2+
True negative	328	423	283	368	364	485	352	467
False negative	42	70	87	125	6	8	18	26
False positive	162	67	136	51	212	91	197	82
True positive	61	33	88	50	11	9	27	19
Sensitivity (%) (95 % CI)	59 (49–69)	32 (23–42)	50 (43–58)	29 (22–36)	65 (38–86)	53 (28–77)	60 (44–74)	42 (28–58)
Specificity (%) (95 % CI)	67 (62–71)	86 (83–89)	68 (63–72)	88 (84–91)	63 (59–67)	84 (81–87)	64 (60–68)	85 (82–88)
PPV (%) (95 % CI)	27 (22–34)	33 (24–43)	39 (33–46)	50 (39–60)	5 (2–9)	9 (4–16)	12 (8–17)	19 (12–28)
NPV (%) (95 % CI)	89 (85–92)	86 (82–89)	76 (72–81)	75 (71–78)	98 (97–99)	98 (97–99)	95 (92–97)	95 (92–97)

*Note*: *PPV* Positive Predictive Value, *NPV* Negative Predictive Value

Using distribution-based cut-points produced generally poorer agreement. 175 children (29 %) were below the “borderline” cut-point in at least one domain. For this outcome, the sensitivity of the NDDS at the 1+ cut-point was 50 % and the specificity 68 %. 45 children (7.6 %) were below at least one “extremely low” cut-point. The sensitivity and specificity in this case were 60 % and 64 %, respectively (see Table 2).

#### Group B (children 4 to 6 years of age)

Seven children (3.2 %) had incomplete or invalid results on one or more instruments, and were excluded from the analysis. Of the remaining 211 children, 40 (19 %) met norms-based criteria for mild delay. At the 1+ cut-point, the NDDS had a sensitivity of 68 % and a specificity of 63 %. For the adjusted outcome, there were 57 cases (27 %). Sensitivity was 60 % and specificity 63 %.

Twelve children (5.7 %) met norms-based criteria for severe delay. The sensitivity of the NDDS was 67 % and the specificity 58 %. Using the adjusted measure produced a prevalence of 8.1 % (17 of 211), a sensitivity of 65 %, and a specificity of 59 % at the 1+ cut-point on the NDDS; (see Table 3).

**Table 3:** Agreement between NDDS and composite indicators of delay for children over 3 (Group B; *n* = 211)

	Mild delay				Severe delay			
	Published norms		Distribution-based cut-points		Published norms		Distribution-based cut-points	
	1+	2+	1+	2+	1+	2+	1+	2+
True negative	107	143	97	130	116	162	114	158
False negative	13	25	23	38	4	6	6	10
False positive	64	28	57	24	83	37	80	36
True positive	27	15	34	19	8	6	11	7
Sensitivity (%) (95 % CI)	68 (51–81)	38 (23–54)	60 (46–72)	33 (21–47)	67 (35–90)	50 (21–79)	65 (38–86)	41 (18–67)
Specificity (%) (95 % CI)	63 (70–55)	84 (89–77)	63 (71–55)	84 (90–78)	58 (65–51)	81 (87–75)	59 (66–51)	81 (87–75)
PPV (%) (95 % CI)	30 (21–40)	35 (21–51)	37 (27–48)	44 (29–60)	9 (4–17)	14 (5–28)	12 (6–21)	16 (7–31)
NPV (%) (95 % CI)	89 (94–82)	85 (90–79)	81 (87–73)	77 (83–70)	97 (99–92)	96 (99–92)	95 (98–89)	94 (97–89)

For severe delay, all PPVs were under 20 %, implying a low probability that a child with a positive screen will meet reference criteria. In keeping with the higher prevalence, PPVs for moderate delay were higher, but still under 50 %. Using the alternative 2+ cut-point raised specificities to 81 %-84 %, but reduced sensitivities to 33 %-50 %.

## Discussion

For screening purposes, it is generally recommended that sensitivity exceed 80 % and specificity 90 % [27]. Given the challenges of screening for developmental delay, lower thresholds (sensitivity of 70 %, specificity of 80 %) have been suggested in this context [28, 29]. The NDDS, however, did not meet either set of criteria. On this basis, we cannot recommend that the NDDS be used on its own for identification of developmental delay in community or population-based settings. Our results are generally consistent with those of Dahinten and Ford [17] who reported 69 % specificity at the −2 SD cut-point on the BSID-II (sensitivity was 100 %, but only 3 cases were identified). Nagy et al. [18] reported much better accuracy (sensitivity 83 %, specificity 95 %), but the criterion measure used in this study was also a parent-reported instrument [18]. Currie et al. reported sensitivity and specificity at the 1+ NDDS threshold to be 75 % and 78 %, respectively, and at the two flag rule, 75 % and 96 %, respectively [16]. As noted previously however, the sample size for this study was very small (*n* = 31), with only 4 children identified with delay. Moreover, the sample was drawn from a high-risk clinical referral group.

The test-retest reliability of the NDDS was also moderate. The retest took place after the clinical assessment, however, and parents of infants and young children (Group A) were often directly involved in the administration of the BSID-III (especially parents of children under 18-months). Parents’ answers on the NDDS retest could therefore have been influenced by what they observed during testing. Especially in young children, it is also conceivable that new behaviours might be observed in a two-week period. It is possible to test whether the latter factor influenced change in parental reporting on the NDDS between test and retest by comparing the proportion of scores that increased (the number of flags indicating delay increased across administrations) versus those that decreased (indicating improvement in development). We found no clear differences in the direction of NDDS changes, however.

As our results illustrate, the validation of measures of developmental delay is difficult, owing to many limitations and challenges in the field. For example, there are numerous possible sources of disagreement beyond faults in the measure being evaluated. While we chose validated, widely-used instruments, there are no definitive, gold standard measures for the identification of ‘developmental delay’. In the case of the NDDS, however, other concerns are evident. First, a reading of items suggests that there is variation across the 13 age bands, resulting in implicit weighting of different domains. The variation in the number of items is another possible issue; endorsement of one item out of 14 on one age band may represent a different threshold than the same score on a version with 22 items. Finally, the NDDS age bands are very wide. The same items and thresholds are used for all 3-year-old children, for example, but substantial development can occur over this year.

Our results have important implications for policy and practice. The NDDS is currently used in a variety of settings to facilitate the identification of developmental delay. Evidence, however, does not support its use as the sole screening measure in any setting. Recommendations for Ontario’s 18-month enhanced well-baby visit [13–15] are to use the NDDS as part of a more comprehensive assessment involving use of other tools (e.g., Rourke Well Baby Record; [30]), and this may be more appropriate. The instrument’s systematic examination of milestones could help initiate discussions with parents and suggest areas for investigation. Given its poor agreement with reference measures, however, we suggest that caution is warranted. If the NDDS is used, it should probably be completed with the assistance of a trained administrator, and its usefulness should be monitored. This might be done, for example, by using administrative data to examine predictive validity.

### Limitations

We evaluated the NDDS in a convenience sample drawn from a single geographical area, and our participating parents were somewhat better-educated than the national average. Although the NDDS consists of 13 separate sets of items, our sample was not large enough for us to evaluate the validity of individual versions. There are also no consensus gold standards for the identification of developmental delay, and the limited age range covered by our primary reference (the BSID-III) obliged us to use different instruments for older children. Given these limitations, independent replication of these results would be valuable.

## Conclusions

The modest test-retest reliability and generally poor agreement with criterion measures leads us to conclude that the NDDS should not be used on its own for the purposes of screening in 1 month to 6 year old children. At the same time, it is important to consider that reference instruments are themselves imperfect. Development is continuous and complex, and, except for clear cases of severe delay, it may be very difficult to construct an instrument relying solely on parental report that will accurately identify children who would benefit from an intervention. Longitudinal data, which make it possible to compare a screen with later health and development, may offer the best prospects in this regard.

## References

[1] Hertzman C, Clinton J, Lynk A, Society CP, Years E, Force T (2011). Measuring in support of early childhood development. Paediatr Child Health.

[2] Baker M (2011). Innis Lecture: Universal early childhood interventions: what is the evidence base?. Can J Econ.

[3] Center on the Developing Child at Harvard University. The Foundations of Lifelong Health Are Built in Early Childhood; 2010. Available: http://developingchild.harvard.edu/resources/the-foundations-of-lifelong-health-are-built-in-early-childhood/. (Accessed 2014 Feb. 4).

[4] Shevell M, Ashwal S, Donley D (2003). Practice parameter: evaluation of the child with global developmental delay: report of the Quality Standards Subcommittee of the American Academy of Neurology and The Practice Committee of the Child Neurology Society. Neurology.

[5] American Academy of Pediatrics, Committee on Children with Disabilities (2001). Developmental surveillance and screening of infants and young children. Pediatrics.

[6] Boyle CA, Decouflé P, Yeargin-Allsopp M (1994). Prevalence and health impact of developmental disabilities in US children. Pediatrics.

[7] Rosenberg SA, Zhang D, Robinson CC (2008). Prevalence of developmental delays and participation in early intervention services for young children. Pediatrics.

[8] McCain MN, Mustard JF (1999). Reversing the real brain drain. The early years study, final report.

[9] McCain MN, Mustard JF, Shanker S. Early years study 2: putting science into action. Toronto (ON): Council for Early Child Development; 2007. Available: www.councilecd.ca/cecd/home.nsf/pages/EYS2.html (accessed 2014 Feb. 4).

[10] American Academy of Pediatrics (2006). Identifying Infants and Young Children with Developmental Disorders in the Medical Home: An Algorithm for Developmental Surveillance and Screening. Pediatrics.

[11] Williams J, Brayne C (2006). Screening for autism spectrum disorders: what is the evidence?. Autism.

[12] Nipissing District Developmental Screen. Nipissing District Developmental Screen Intellectual Property Association; 2000. North Bay: ndds. Available: www.ndds.ca (accessed 2014 Feb. 4).

[13] Expert Panel on the 18 Month Well-Baby Visit. Getting it right at 18 month. Making it right for a lifetime; 2005. Available: www.children.gov.on.ca/htdocs/English/documents/topics/earlychildhood/getting_it_right_18_months.pdf (accessed 2014 Feb. 4).

[14] Williams R, Clinton J (2011). Getting it right at 18 months: In support of an enhanced well-baby visit. Paediatr Child Health.

[15] Williams R, Clinton J, Price D, et al. Ontario’s Enhanced 18-Month Well-Baby Visit: program overview, implications for physicians. Ontario Medical Review. 2010;23–27.

[16] Currie L, Dodds L, Shea S (2012). Investigation of test characteristics of two screening tools in comparison with a gold standard assessment to detect developmental delay at 36 months: A pilot study. Paediatr Child Health.

[17] Dahinten SV, Ford L. Validation of the Nipissing District Developmental Screen for Use with Infants and Toddlers (Working Paper). Unpublished Report from the Human Early Learning Partnership (HELP); 2004. Available: http://ndds.ca/images/stories/pdfs/2004Dahinten_Nippising.pdf. (Accessed 2014 Feb. 4).

[18] Nagy P, Ryan B, Robinson R, et al. Nipissing Instrument Validation Report, 2001–2002. In Evaluation of Healthy Babies, Healthy Children Program [working paper]. Early Years and Healthy Child Development Branch, Ontario Ministry of Community, Family and Children's Services; 2002.

[19] Bayley N (2006). Bayley Scales of Infant Development.

[20] Henderson SE, Sugden DA, Barnett AL (2007). Movement Assessment Battery for Children-2, Second Edition (Movement ABC-2): Examiner’s manual.

[21] Zimmerman IL, Steiner VG, Pond RE (2002). Preschool Language Scale-4.

[22] Zimmerman IL, Castilleja NF (2005). The role of a language scale for infant and preschool assessment. Ment Retard Dev Disabil Res Rev.

[23] Kaufman AS, Kaufman NL (2004). Kaufman Brief Intelligence Test.

[24] Anderson PJ, De Luca CR, Hutchinson E (2010). Underestimation of developmental delay by the new Bayley-III scale. Arch Pediatr Adolesc Med.

[25] Royston P. fp_plus: Multivariable fractional polynomial models with extensions [Computer software] 2012. London, UK: University College London. Available: www.homepages.ucl.ac.uk/~ucakjpr/stata/fp_plus/xmfp.sthlp (accessed 2013 Nov. 1).

[26] Regression with Stata. Chapter 4: Beyond OLS. UCLA: Statistical Consulting Group. Available: www.ats.ucla.edu/stat/stata/webbooks/reg/chapter4/statareg4.htm (accessed October, 2013).

[27] Streiner DL, Norman GR (1995). Health measurement scales: A practical guide to their development and use.

[28] Glascoe FP, Marks KM, Poon JK, Macias MM (eds.). Identifying and addressing developmental-behavioral problems: a practical guide for medical and non-medical professionals, trainees, researchers and advocates. Nolensville, Tennessee: PEDStest.com; 2013.

[29] Bricker D, Squires J (1989). Low cost system using parents to monitor the development of at-risk infants. J Early Interv.

[30] Rourke L, Godwin M, Rourke J (2009). The Rourke Baby Record Infant/Child Maintenance Guide: do doctors use it, do they find it useful, and does using it improve their well-baby visit records?. BMC Fam Pract.

